# Accelerated heterologous prime-boost adenovirus vector-based SIV vaccine in neonatal rhesus monkeys

**DOI:** 10.1186/1742-4690-9-S2-O48

**Published:** 2012-09-13

**Authors:** J Liu, H Li, M Iampietro, DH Barouch

**Affiliations:** 1 Beth Israel Deaconess Medical Center, Harvard Medical School, Boston, MA, USA

## Background

A pediatric HIV-1 vaccine is required to protect infants against HIV-1 transmission from breastfeeding. Such a vaccine would need to induce protective immunity at mucosal surfaces in neonates as soon as possible after birth. Recombinant adenovirus (rAd) vectors have been shown to elicit potent systemic and mucosal virus-specific immune responses in adult non-human primates and humans but have not previously been studied in detail in infants.

## Methods

Newborn rhesus monkeys were injected intramuscular (i.m.) with 10^11 viral particles of rAd serotype 26 or 35 vectors expressing SIVmac239 Gag. Peripheral blood was collected to determine systemic Gag-specific cellular and humoral immune responses. At week 52, peripheral lymph nodes, bronchoalveolar lavage (BAL) and pinch biopsies of colorectal, duodenal and oral cavity mucosa were collected to evaluate mucosal Gag-specific T lymphocyte responses.

## Results

A single injection of rAd26 encoding SIVmac239 Gag in rhesus monkeys on the day of birth elicited detectable SIV-specific cellular immune responses, but these responses were transient and waned quickly. In contrast, an accelerated heterologous prime-boost regimen involving administration of rAd35 at birth and rAd26 at 4 weeks of life elicited potent and durable Gag-specific cellular and humoral immune responses in neonatal rhesus monkeys, including mucosal responses that remained detectable at one year of age.

## Conclusion

These results suggest the potential of an accelerated heterologous rAd prime-boost regimen as a candidate neonatal HIV-1 vaccine in newborns.

